# Supplementary material for: Transient non-soluble noble metal transport in hydrothermal ore systems
Source: Nat Commun. 2025 Mar 13;16:2521. doi: 10.1038/s41467-025-57740-7 (PMC11906853; doi:10.1038/s41467-025-57740-7)
Supplement: Supplementary file 1 — Supplementary Information [file 41467_2025_57740_MOESM1_ESM.pdf]

Transient non-soluble noble metal transport in hydrothermal ore systems

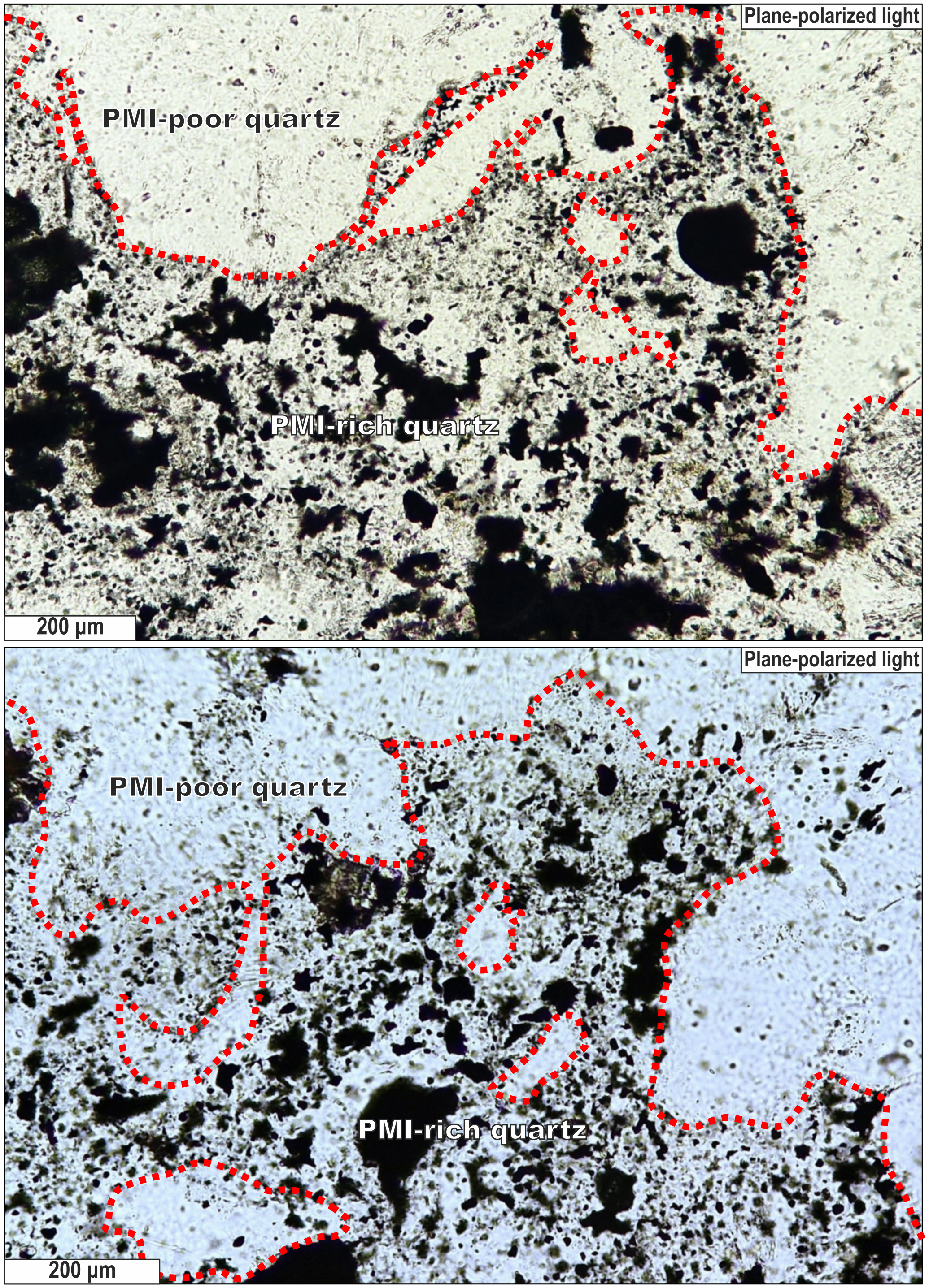

Supplementary Fig. 1. Plane-polarized light photomicrographs showing replacement textures (e.g., “islands”, embayments, lobulated and irregular contacts) indicating that PMI-rich quartz replaces PMI-poor quartz. These textures suggest coupled dissolution-reprecipitation reactions between PMI-poor quartz and hydrothermal fluids carrying PMIs. PMI: Polymineral inclusions.

2. SUPPLEMENTAL FIGURES

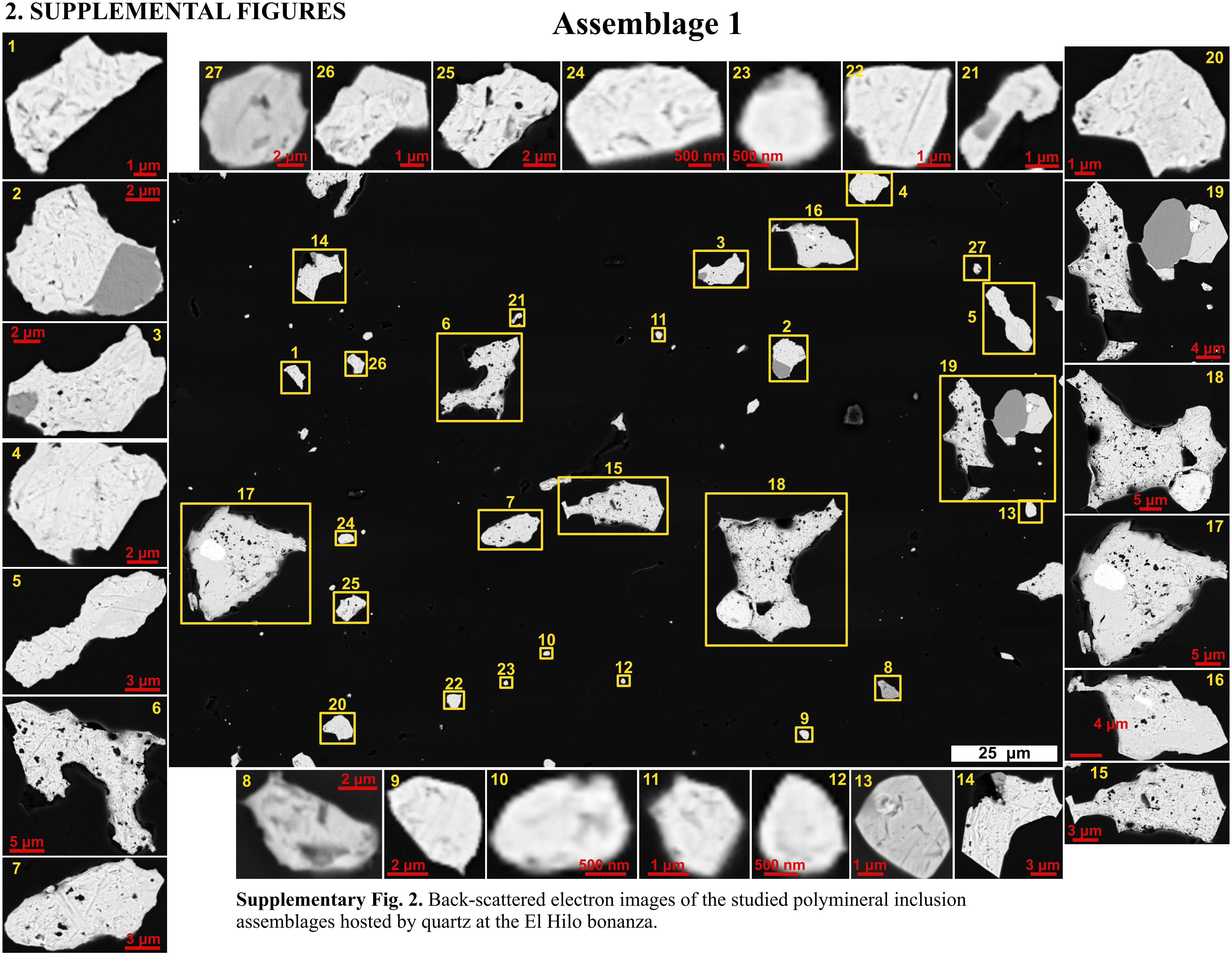

Supplementary Fig. 2. Back-scattered electron images of the studied polymineral inclusion assemblages hosted by quartz at the El Hilo bonanza.

Assemblage 1 (interpretation)

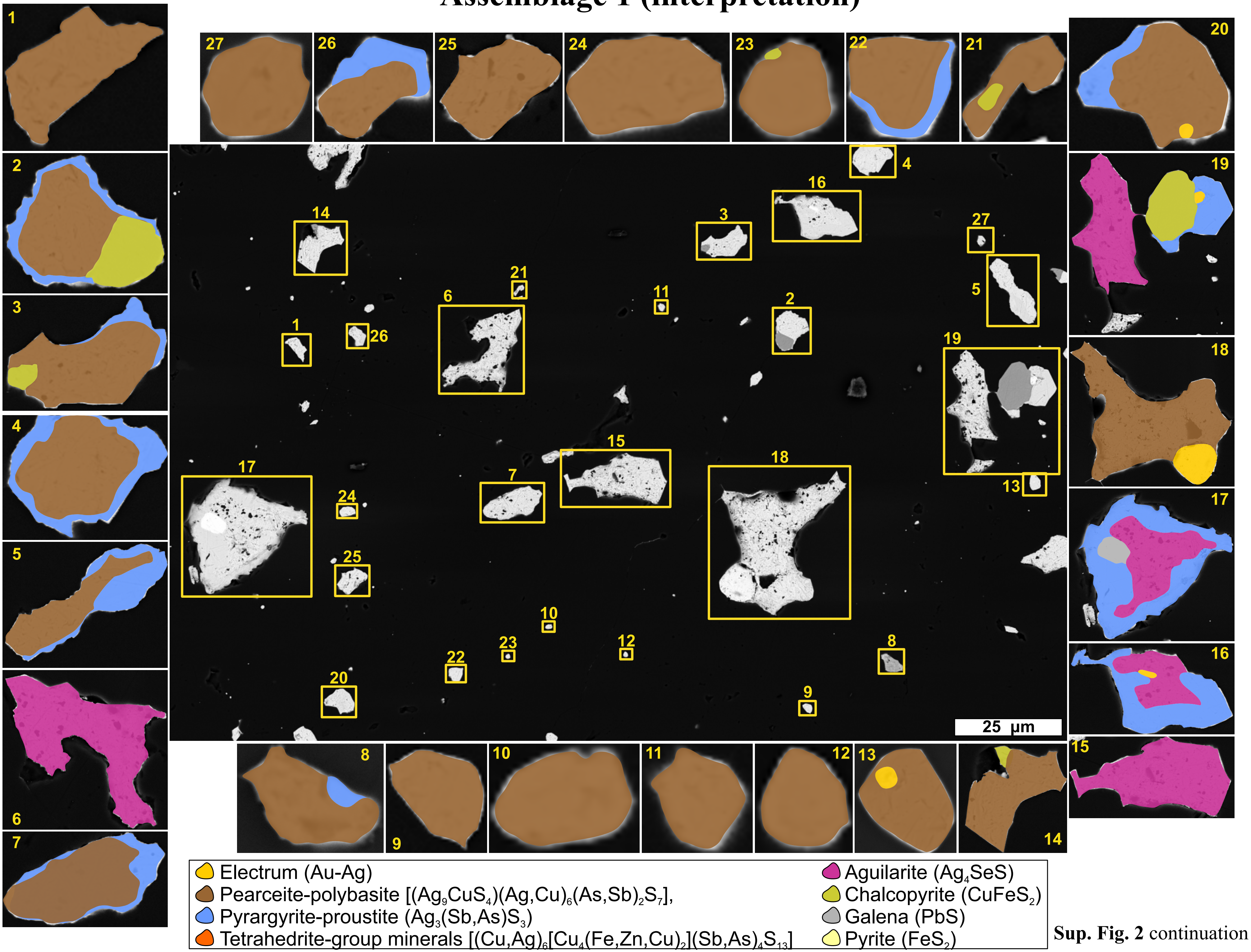

Sup. Fig. 2 continuation

Assemblage 2

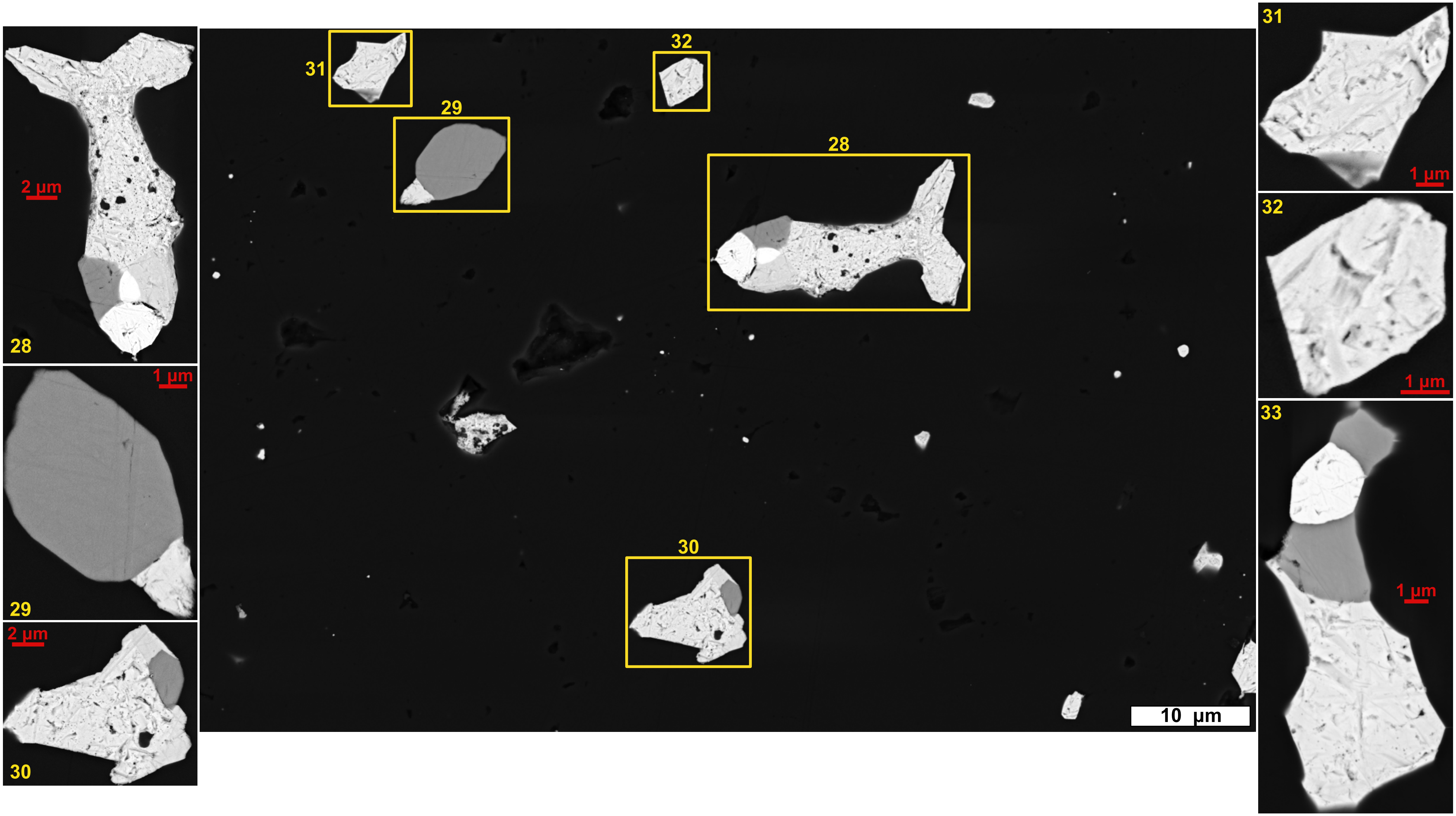

Sup. Fig. 2 continuation

Assemblage 2 (interpretation)

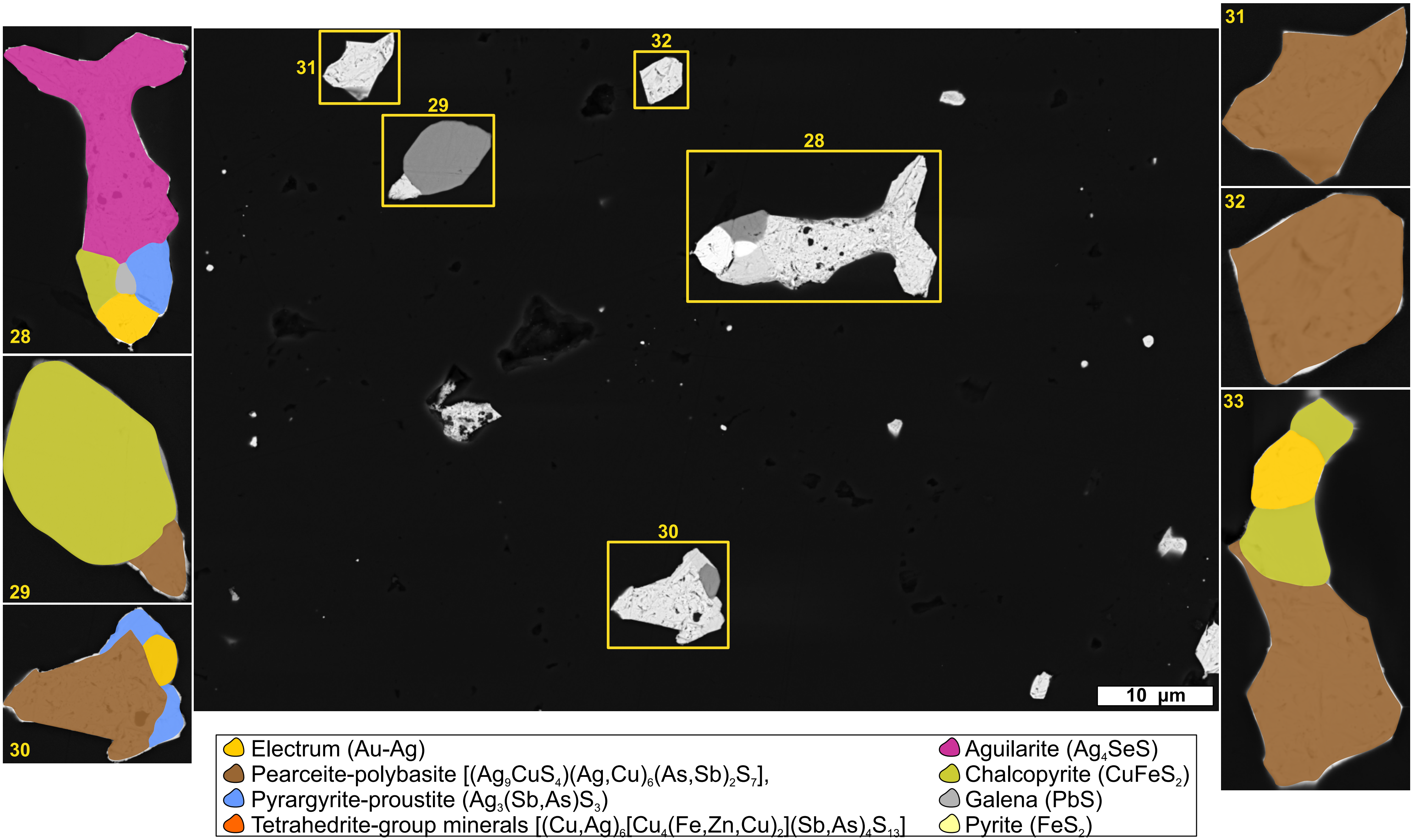

Sup. Fig. 2 continuation

Assemblage 3

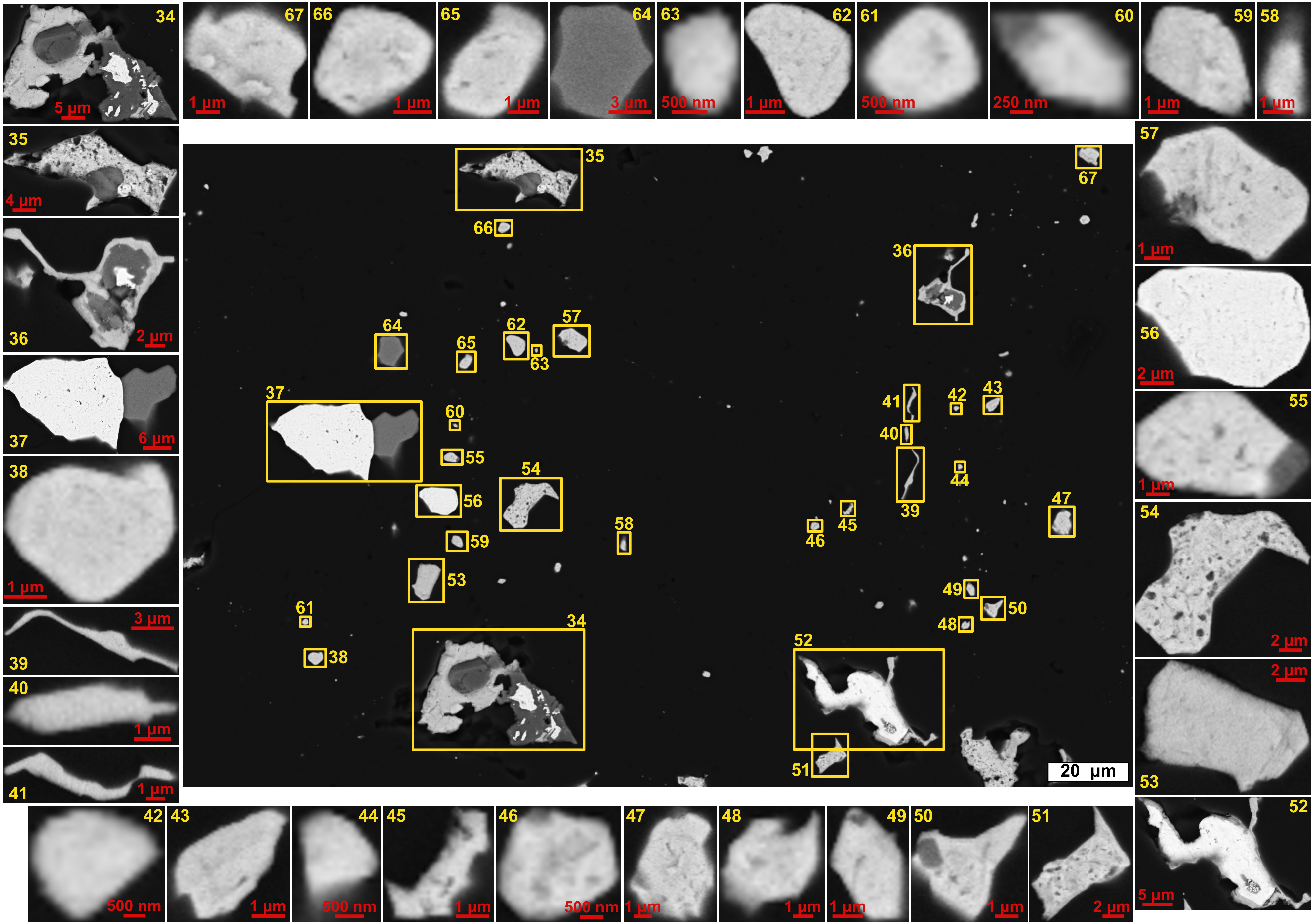

Sup. Fig. 2 continuation

Assemblage 3 (interpretation)

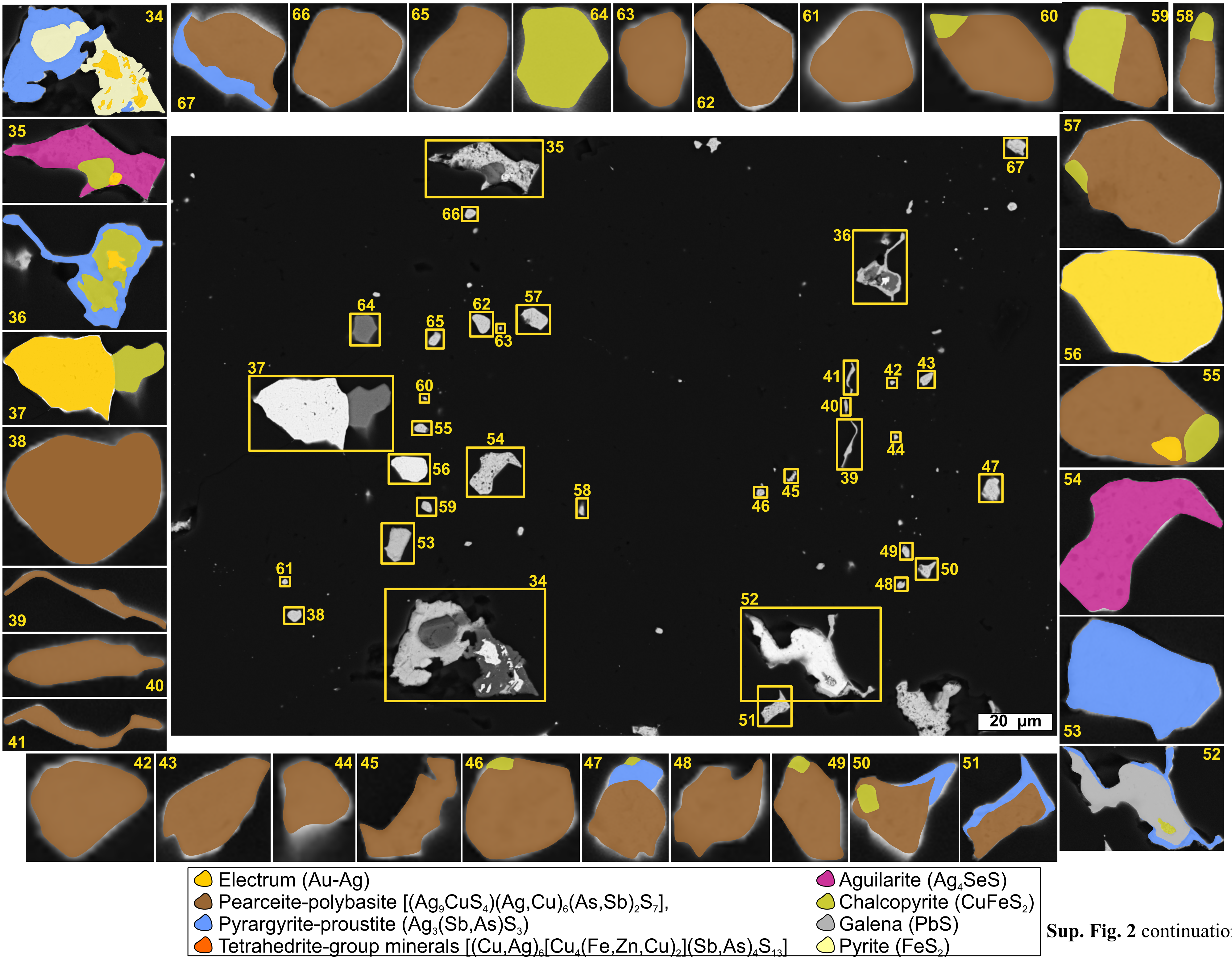

Sup. Fig. 2 continuation

Assemblage 4

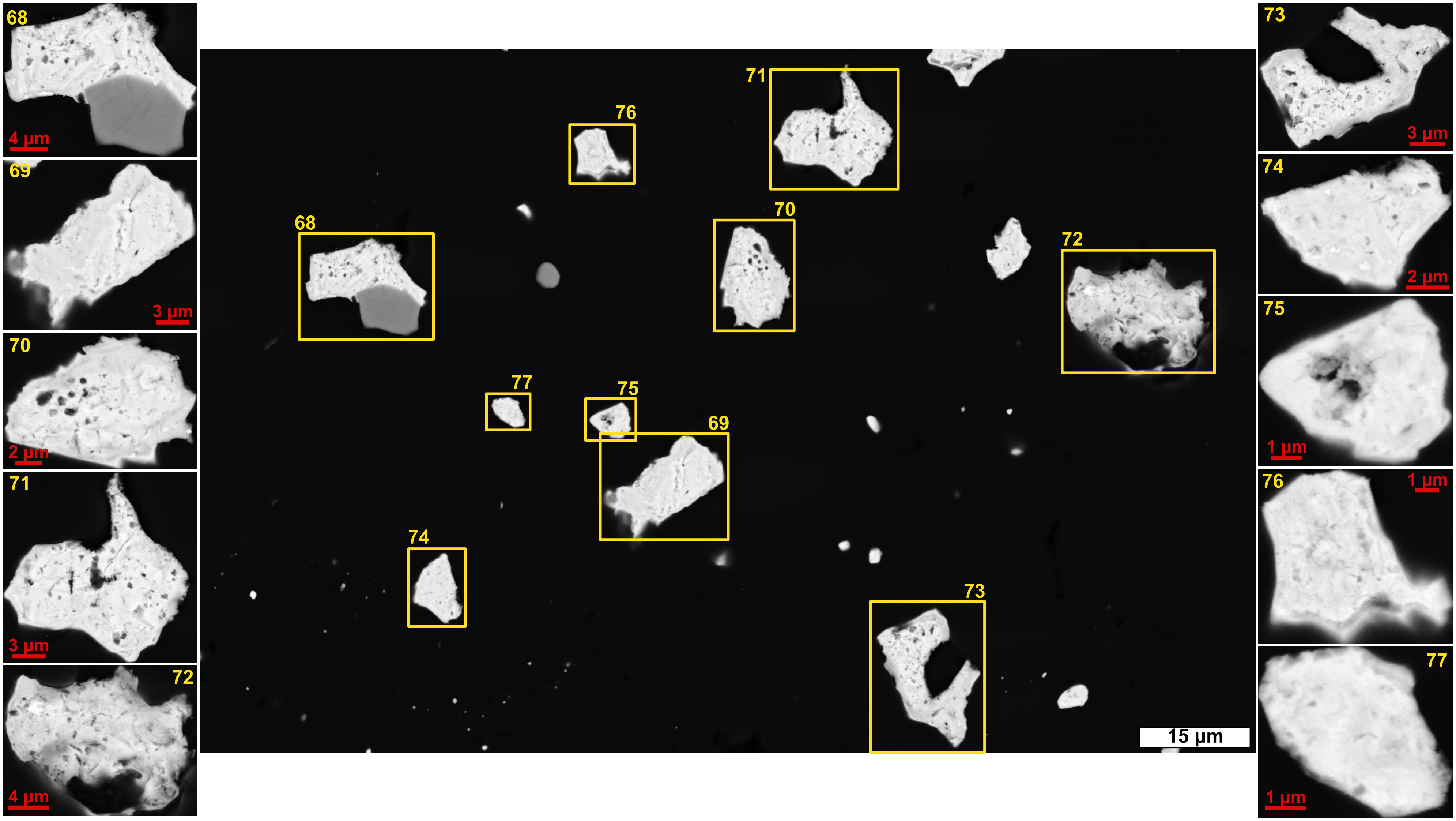

Sup. Fig. 2 continuation

Assemblage 4 (interpretation)

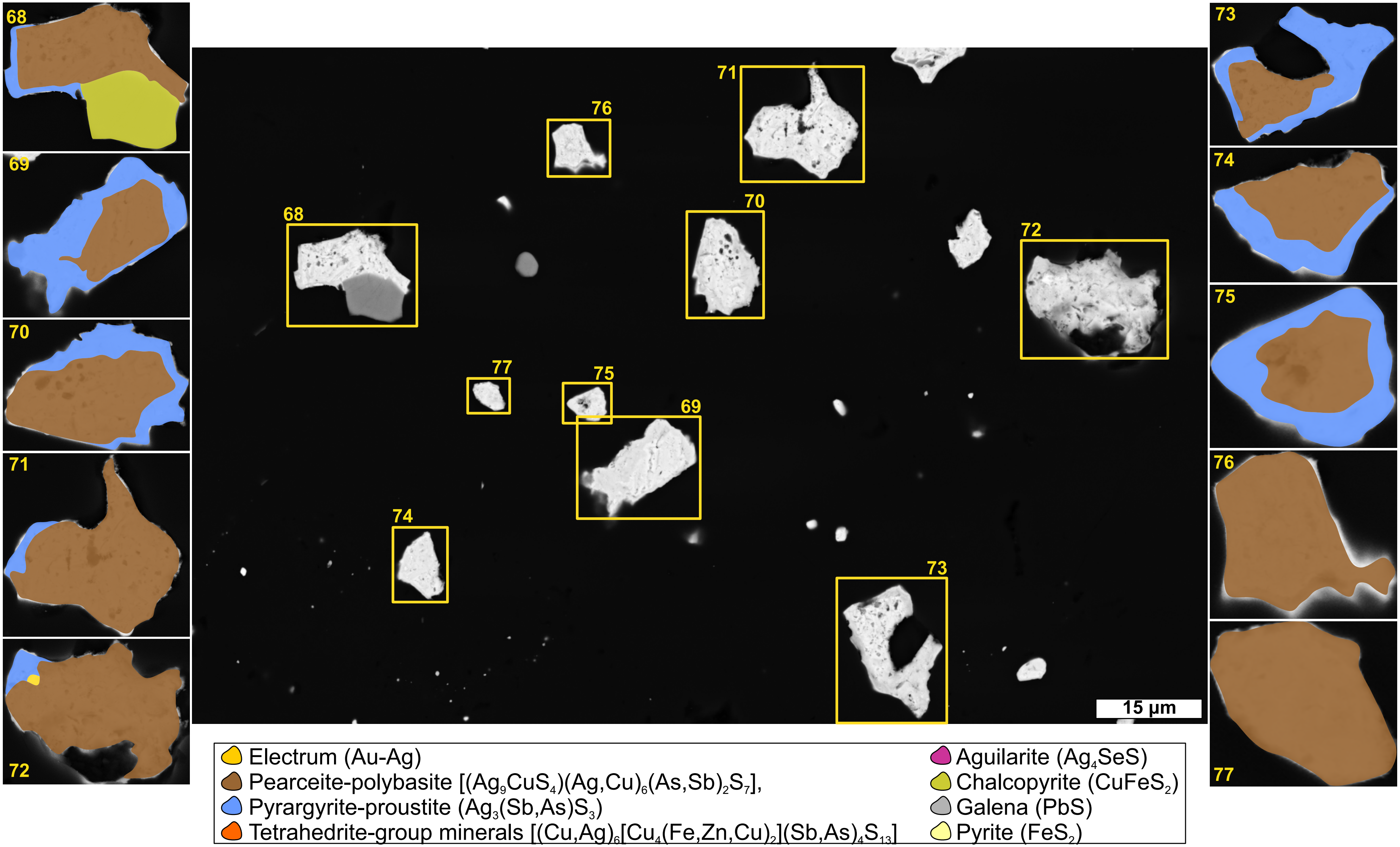

Sup. Fig. 2 continuation

Assemblage 5

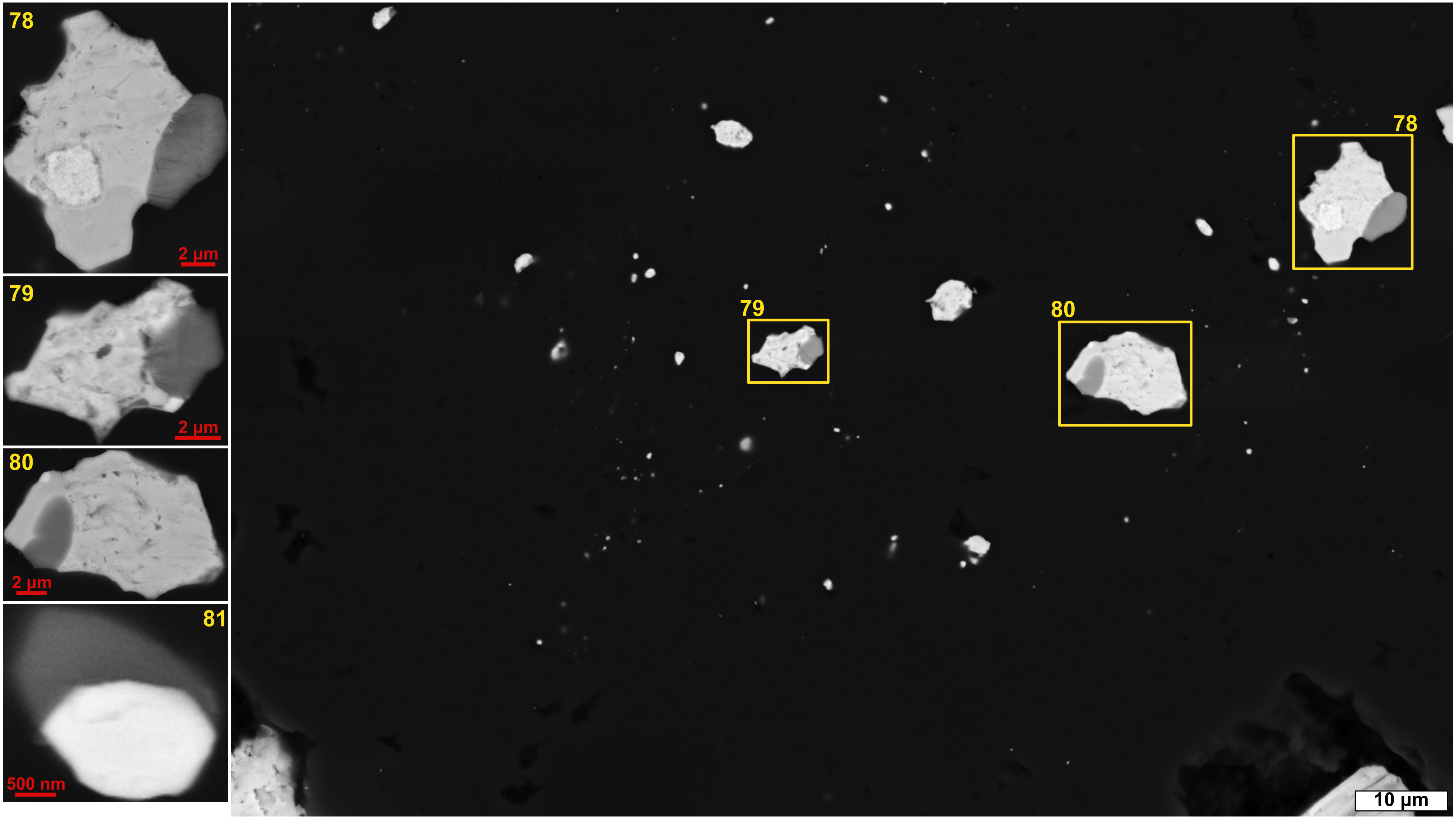

Sup. Fig. 2 continuation

Assemblage 5 (interpretation)

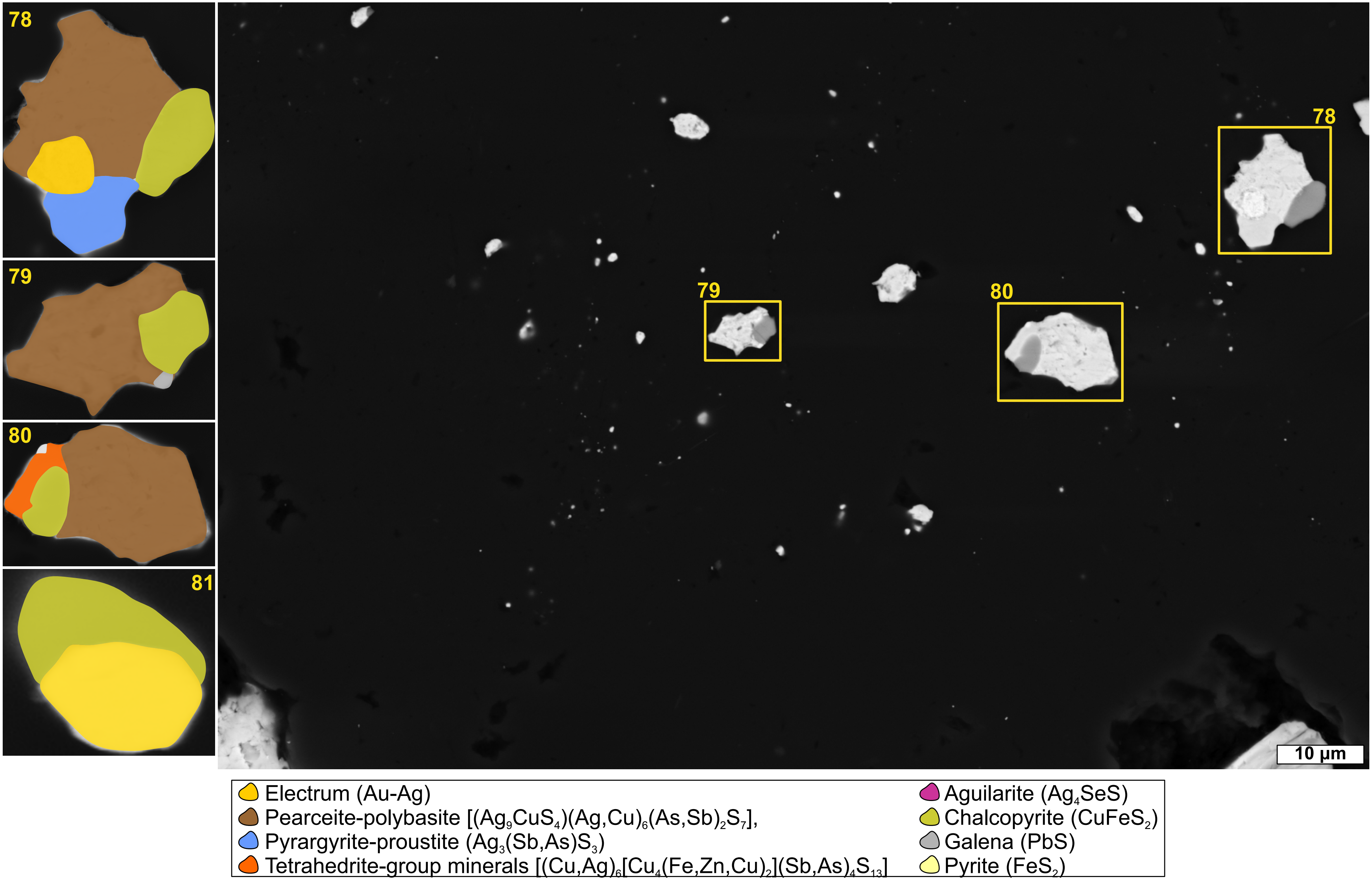

Sup. Fig. 2 continuation

Assemblage 5

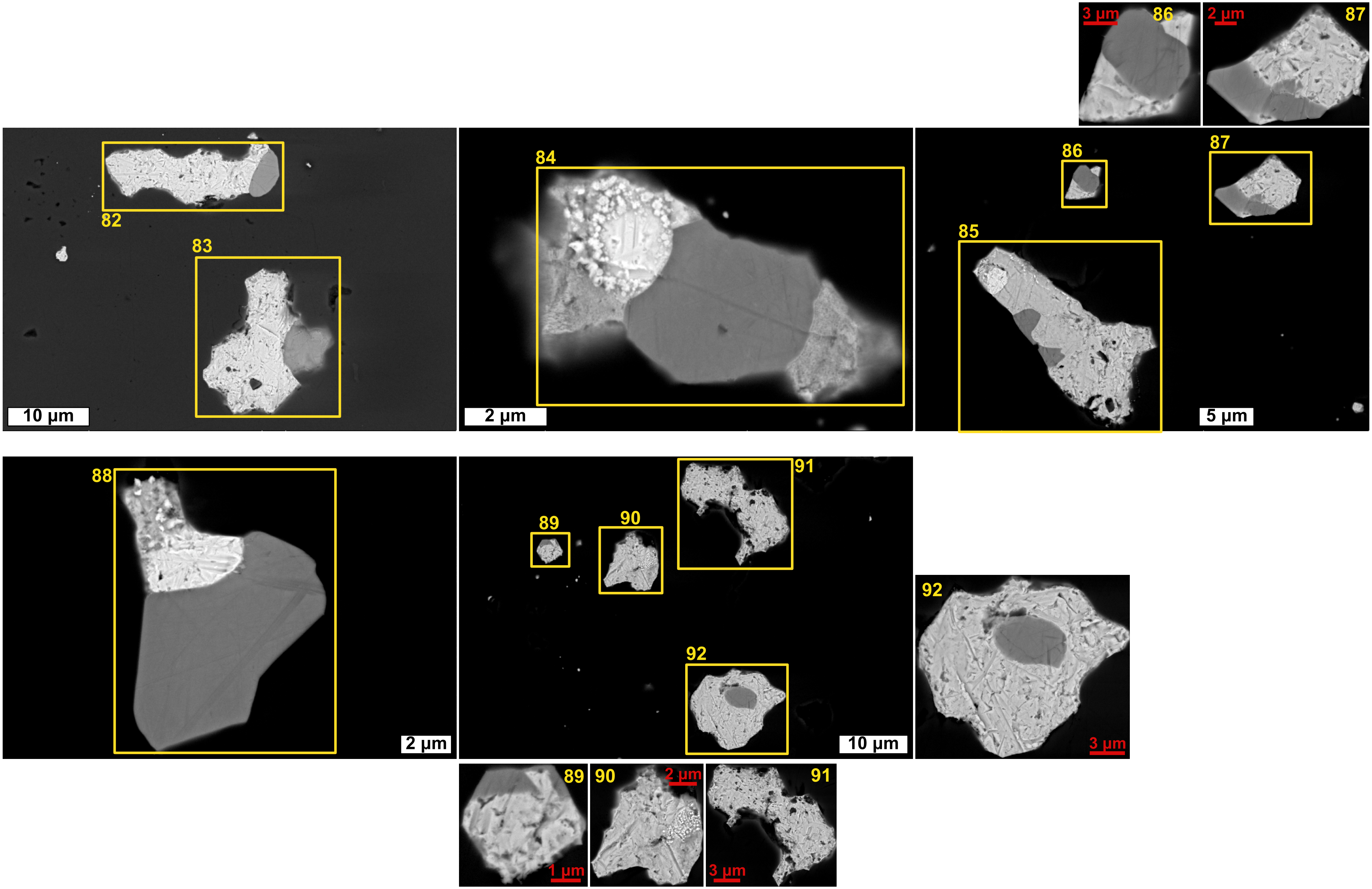

Sup. Fig. 2 continuation

Assemblage 5 (interpretation)

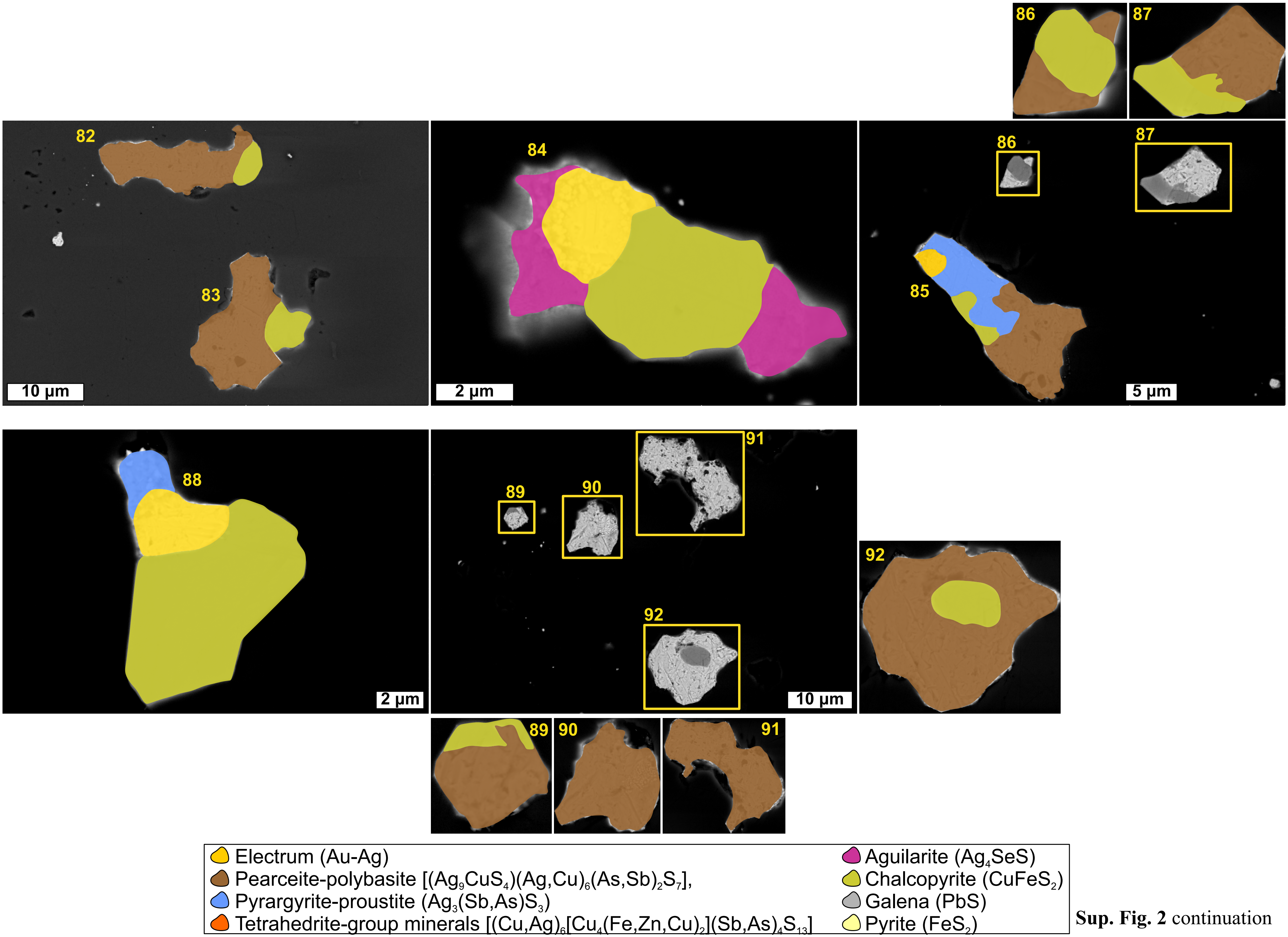

Sup. Fig. 2 continuation

Assemblage 5

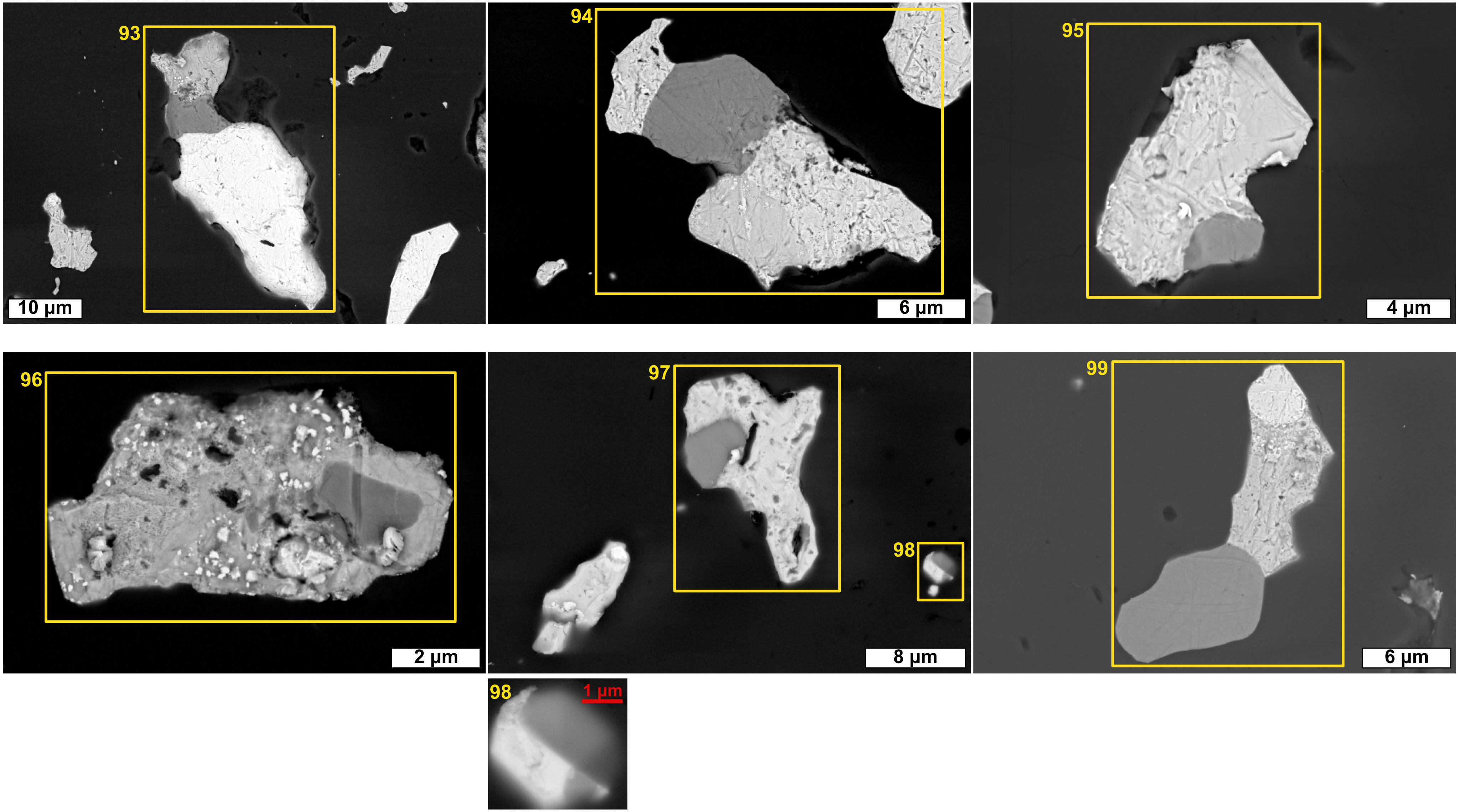

Sup. Fig. 2 continuation

Assemblage 5 (interpretation)

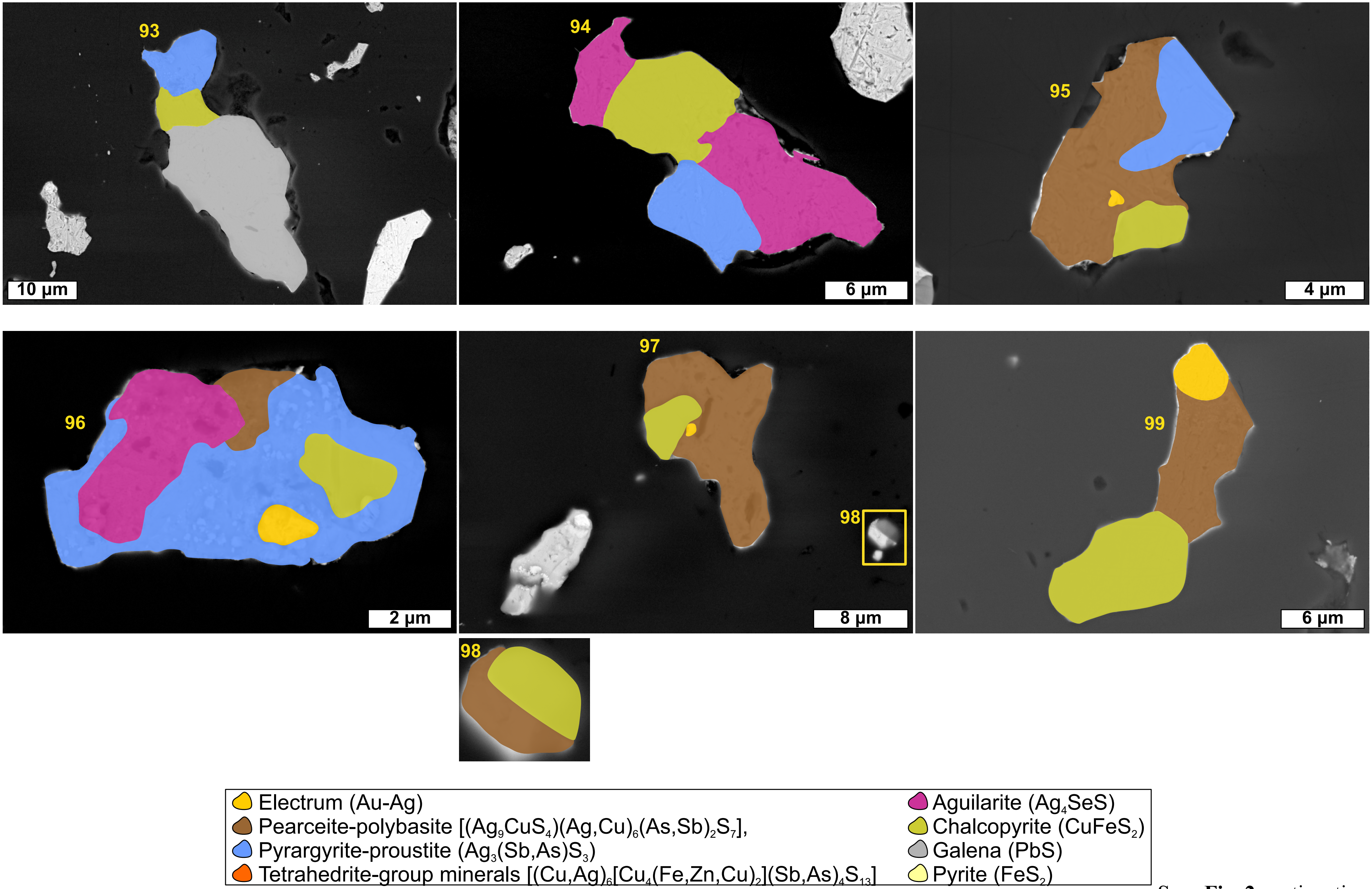

Sup. Fig. 2 continuation

Assemblage 6

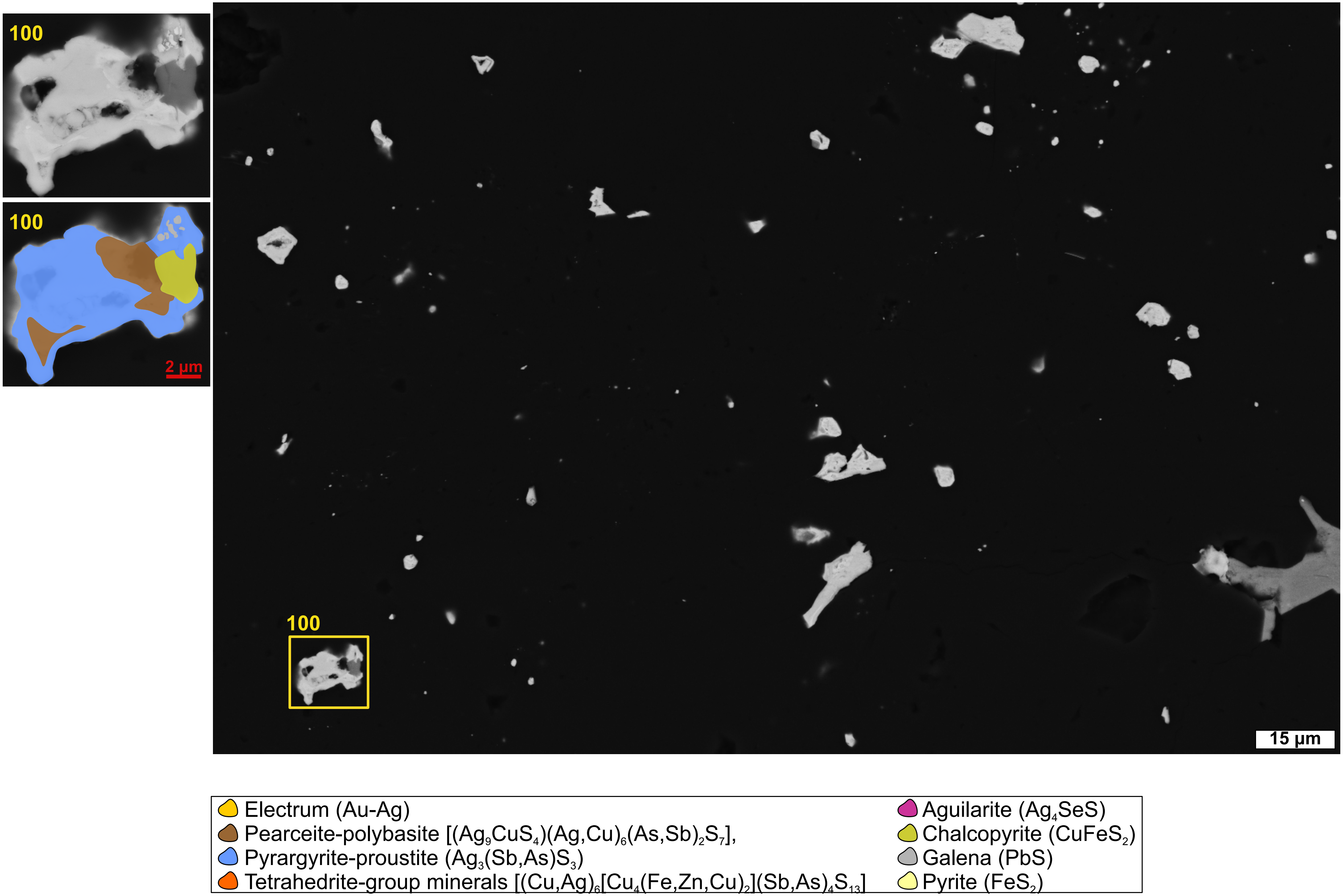

Sup. Fig. 2 continuation

Assemblage 7

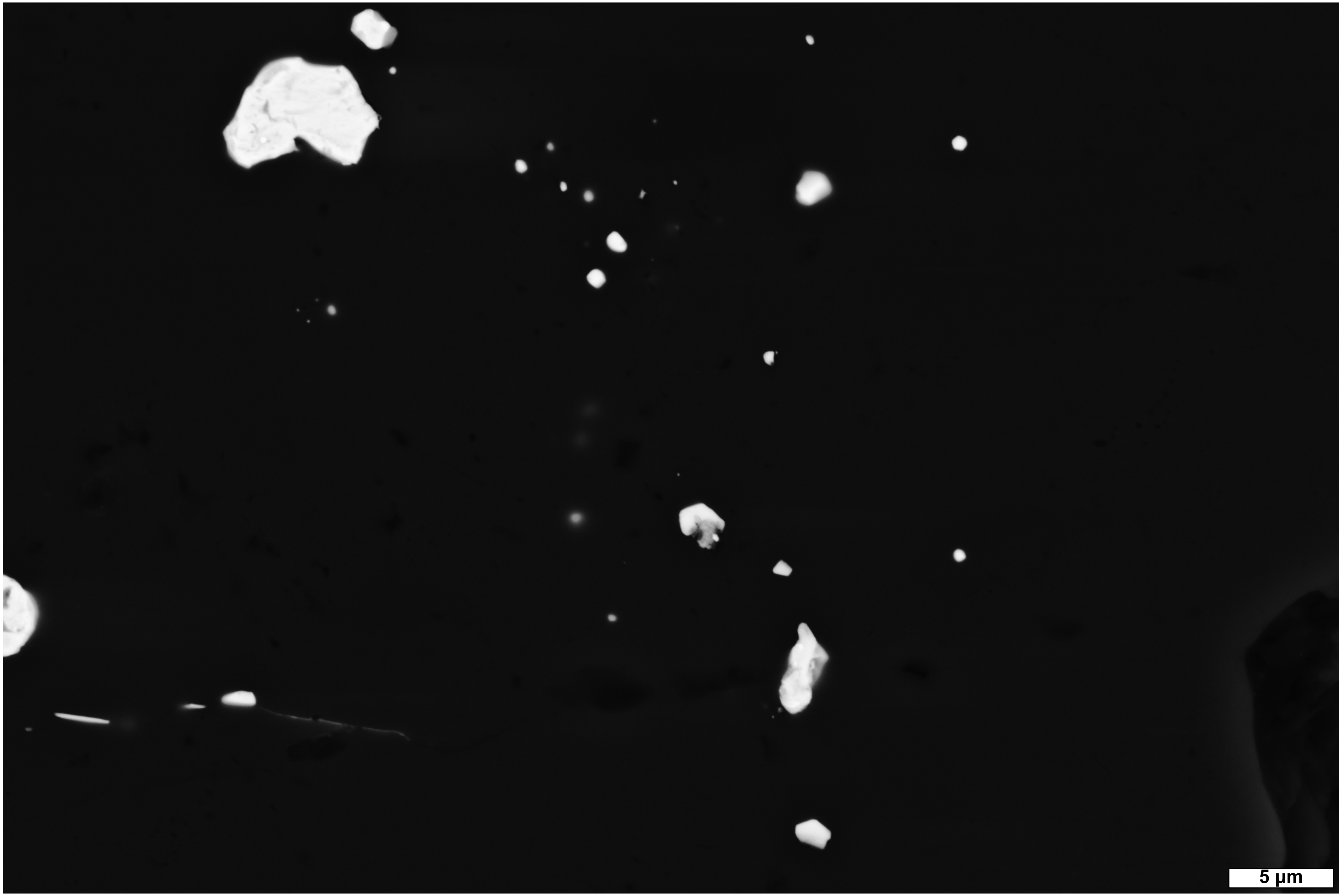

Sup. Fig. 2 continuation

Assemblage 8

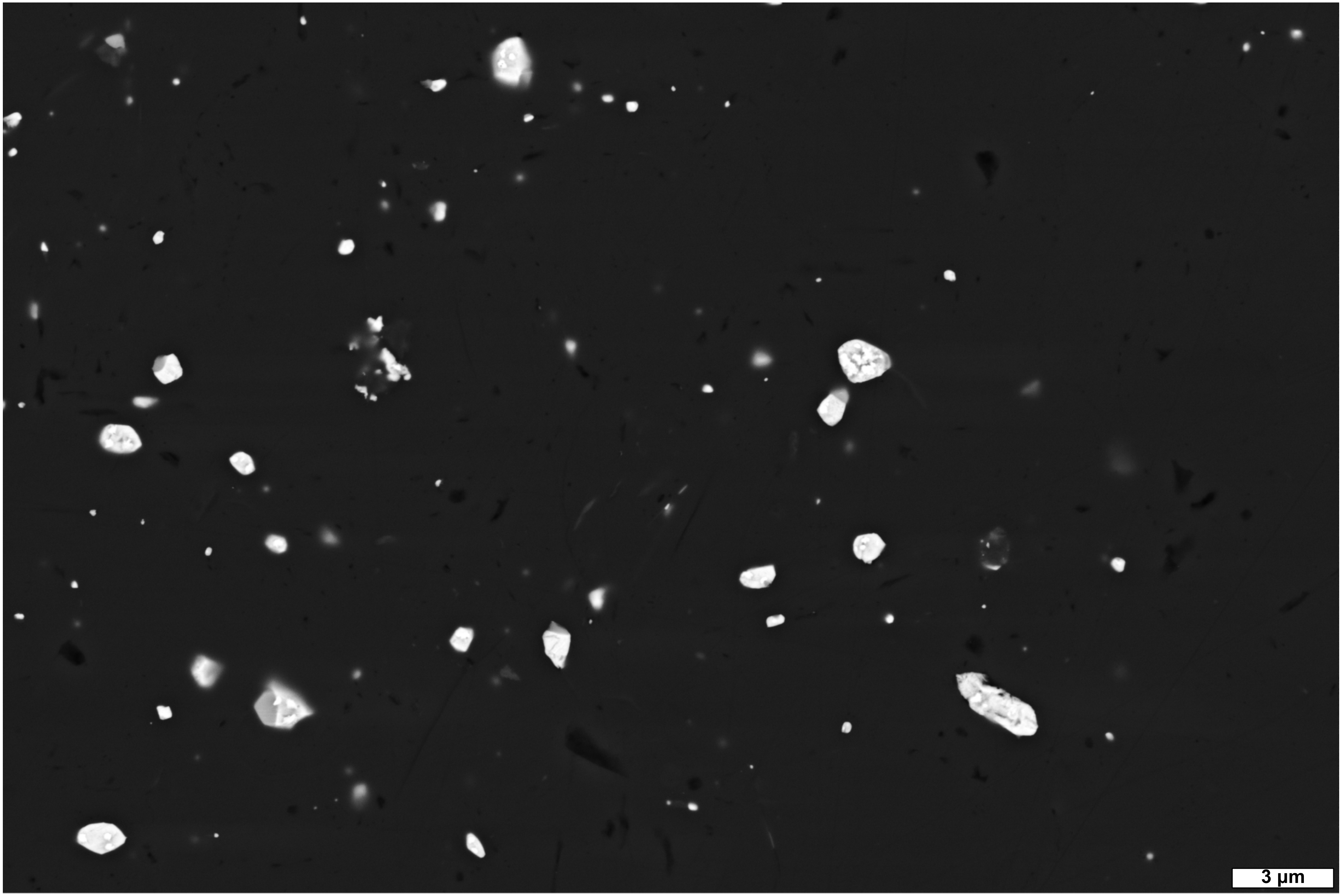

Sup. Fig. 2 continuation

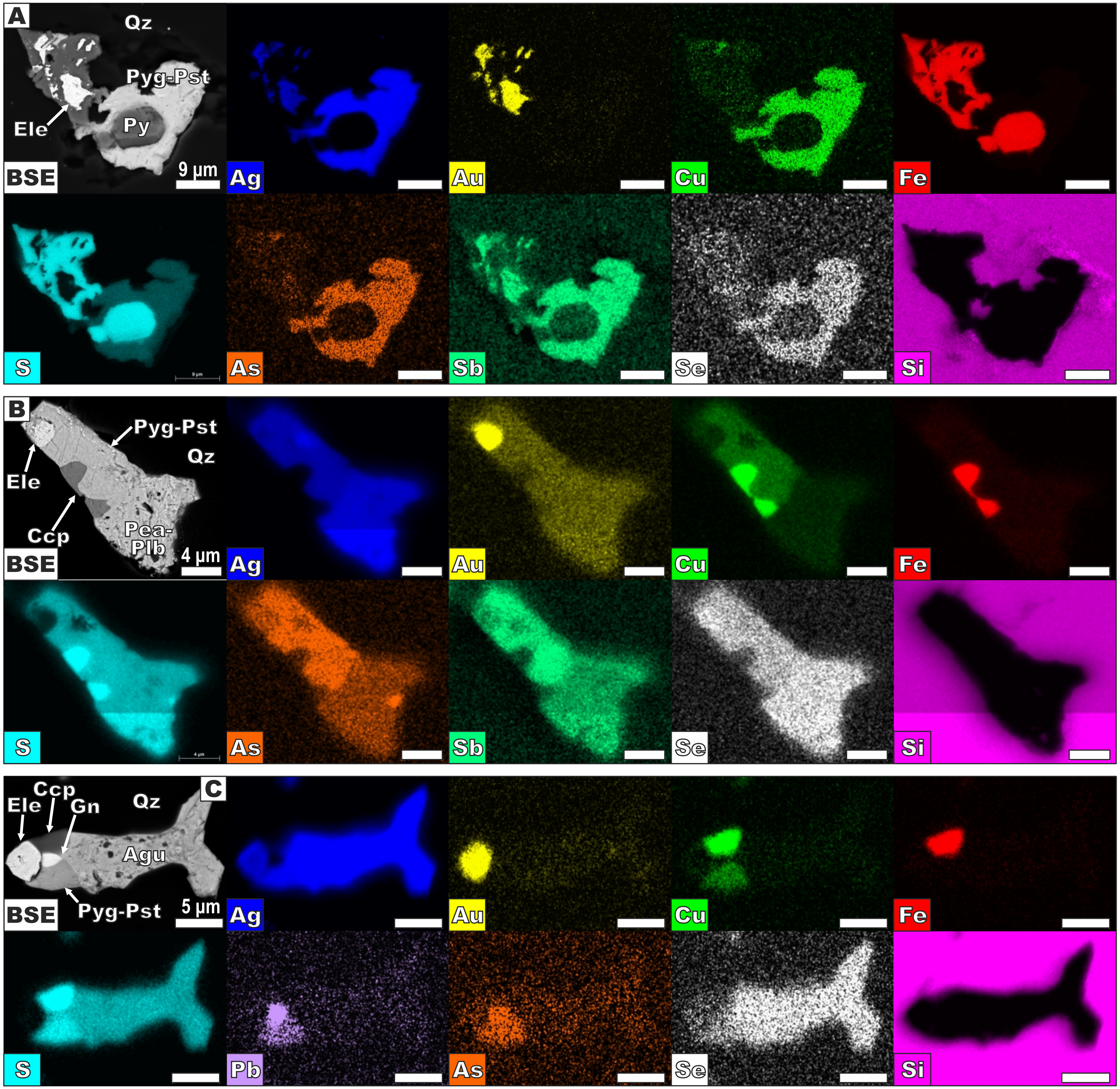

Supplementary Fig. 3. Panels A to C show scanning electron microscopy (SEM) energy-dispersive X-ray (EDX) compositional maps of some polymineral inclusions (PMI). Key: Agu = aguilarite, Ccp = chalcopyrite, Ele = electrum (Au-Ag alloy), Gn = galena, Pea = pearceite, Plb = polybasite, Prs = proustite, Py = pyrite, Pyg = pyrargyrite, Qz = quartz.

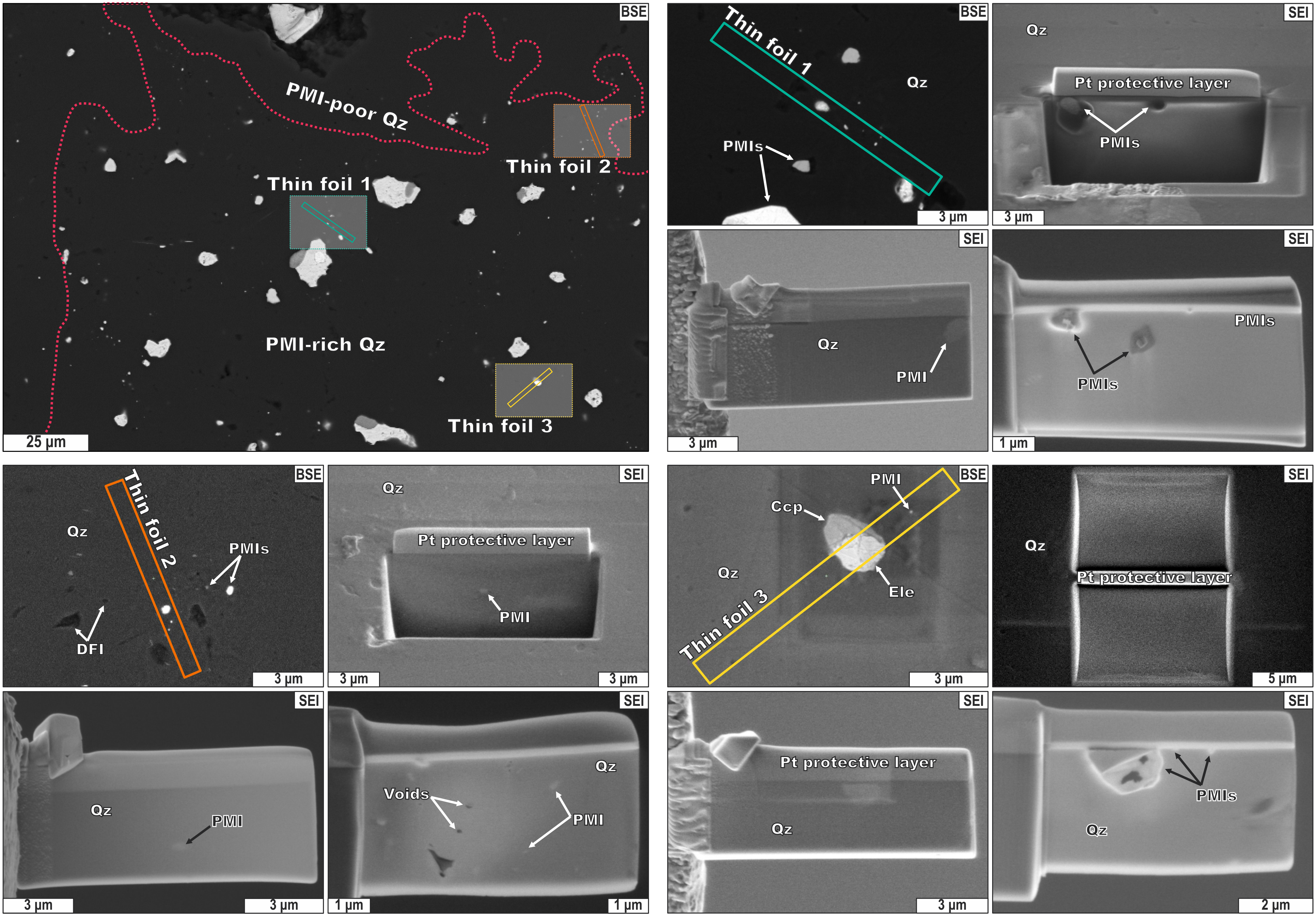

Supplementary Fig. 4. Back-scattered electron (BSE) and secondary electron (SEI) images showing the location and preparation process of the studied thin foils (assemblage 5). See small voids in thin foil 2 that could represent decrepitated fluid inclusions. Key: Ccp = chalcopyrite, Ele = electrum, PMI = polymineral inclusion, Qz = quartz.

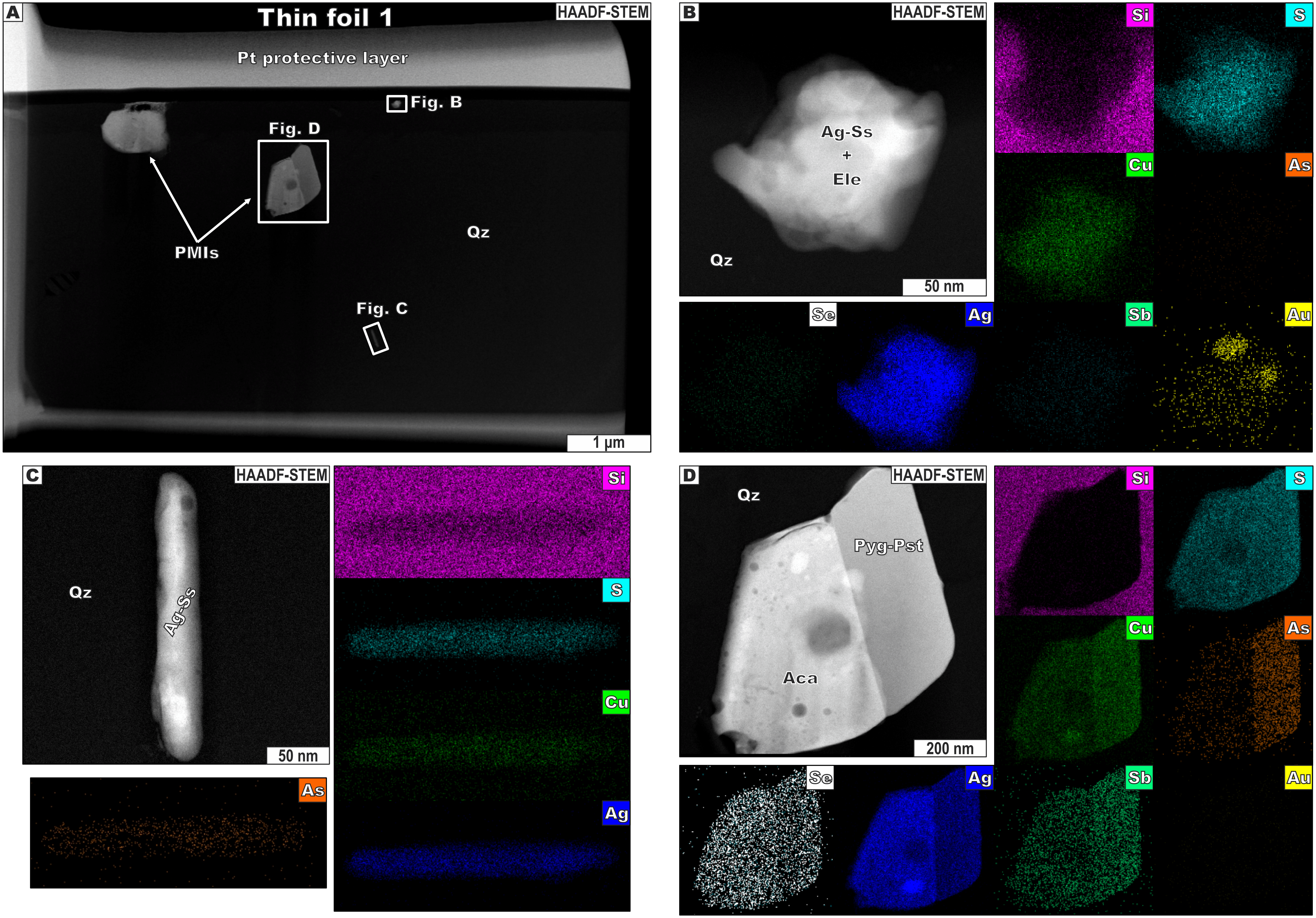

Supplementary. Fig. 5. A. High-angle annular dark-field (HAADF) image of thin foil 1. B-D. Scanning transmission electron microscopy (STEM) HAADF images and STEM compositional maps of polymineral inclusions (PMI) from thin foil 1. Key: Aca = acanthite, Ag-Ss = Ag sulfosalt, Ccp = chalcopyrite, Ele = electrum (Au-Ag alloy), Pea = pearceite, Plb = polybasite, Prs = proustite, Pyg = pyrargyrite, Qz = quartz.

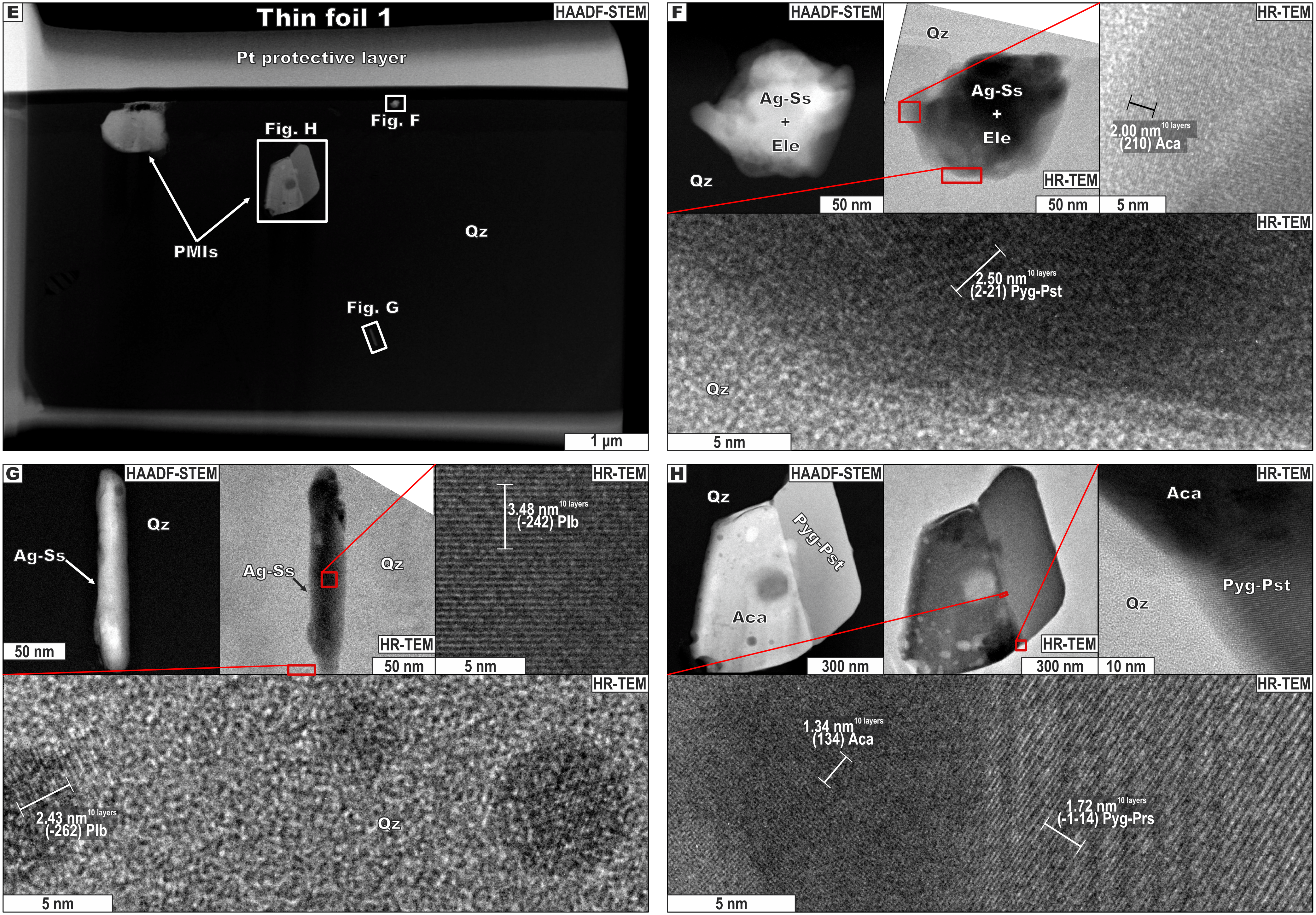

Supplementary Fig. 5. E. High-angle annular dark-field (HAADF) image of thin foil 1. F-H. Scanning transmission electron microscopy (STEM) HAADF images and high-resolution TEM images of polymineral inclusions (PMI) from thin foil 1. Key: Aca = acanthite, Ag-Ss = Ag sulfosalt, Ccp = chalcopyrite, Ele = electrum (Au-Ag alloy), Pea = pearceite, Plb = polybasite, Prs = proustite, Pyg = pyrargyrite, Qz = quartz.

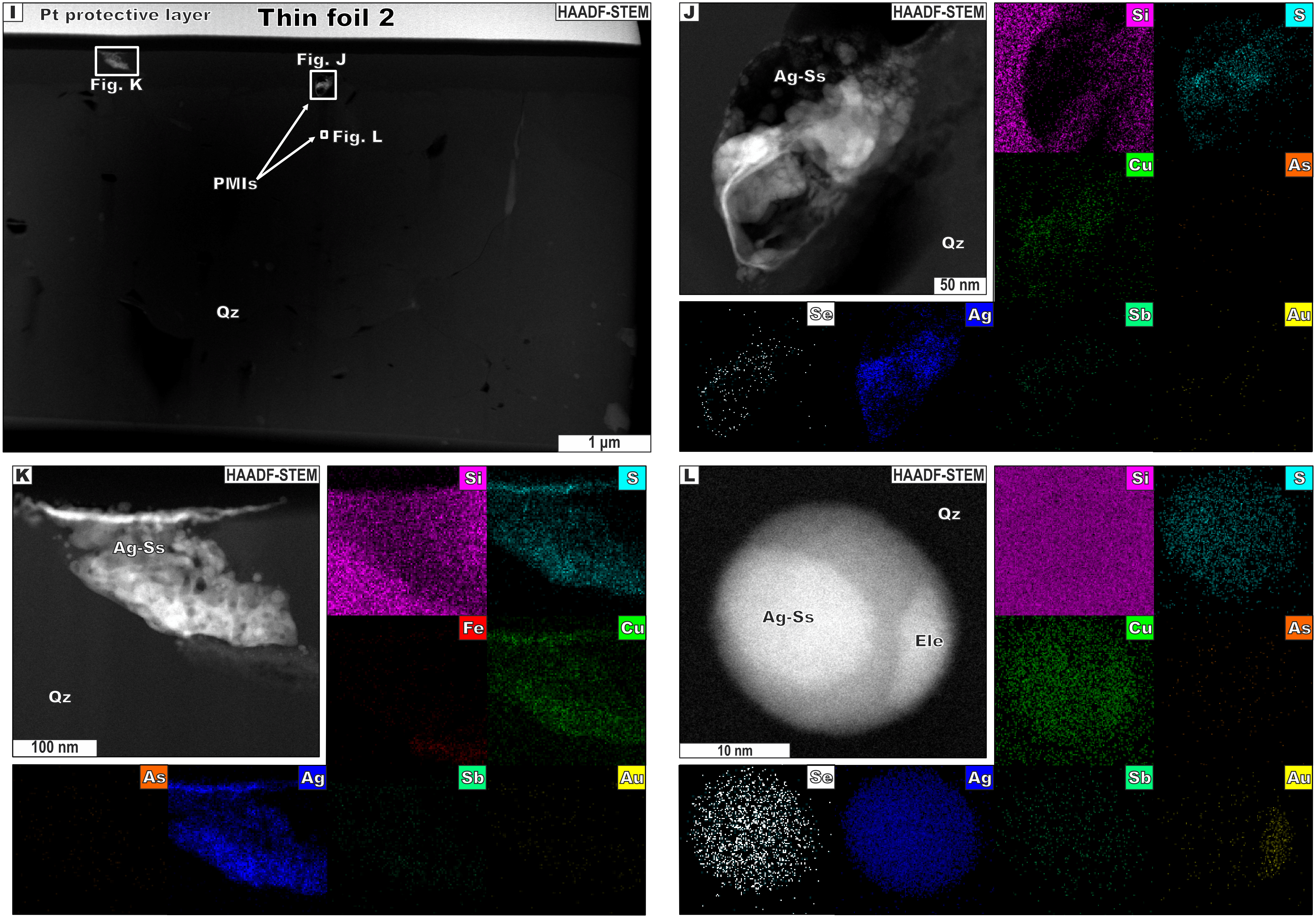

Supplementary Fig. 5. I. High-angle annular dark-field (HAADF) image of thin foil 2. J-L. Scanning transmission electron microscopy (STEM) HAADF images and STEM compositional maps of polymineral inclusions (PMI) from thin foil 2. Key: Aca = acanthite, Ag-Ss = Ag sulfosalt, Ccp = chalcopyrite, Ele = electrum (Au-Ag alloy), Pea = pearceite, Plb = polybasite, Prs = proustite, Pyg = pyrargyrite, Qz = quartz.

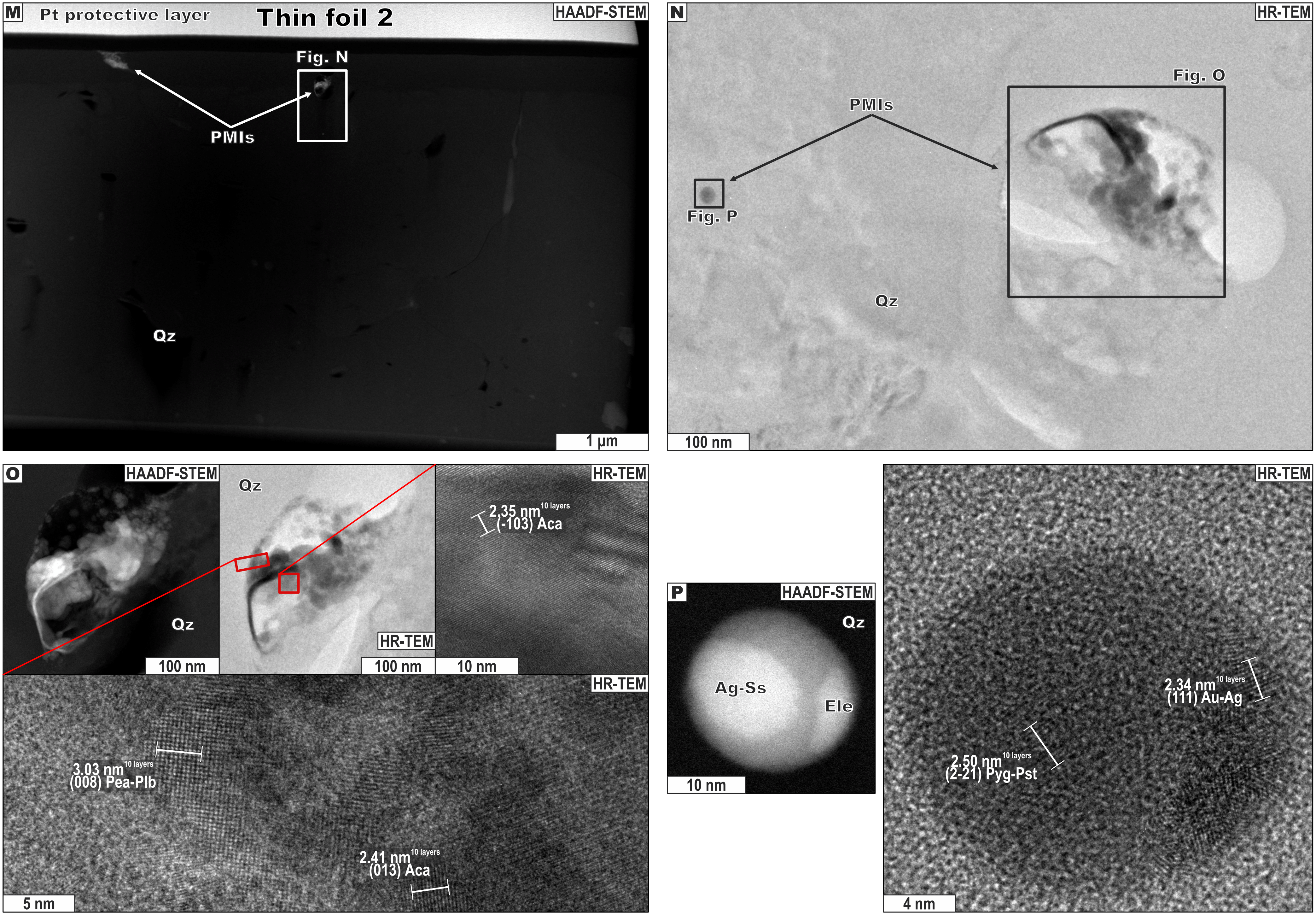

Supplementary Fig. 5. M. High-angle annular dark-field (HAADF) image of thin foil 2. N-P. Scanning transmission electron microscopy (STEM) HAADF images and high-resolution TEM images of polymineral inclusions (PMI) from thin foil 2. Key: Aca = acanthite, Ag-Ss = Ag sulfosalt, Ccp = chalcopyrite, Ele = electrum (Au-Ag alloy), Pea = pearceite, Plb = polybasite, Prs = proustite, Pyg = pyrargyrite, Qz = quartz.

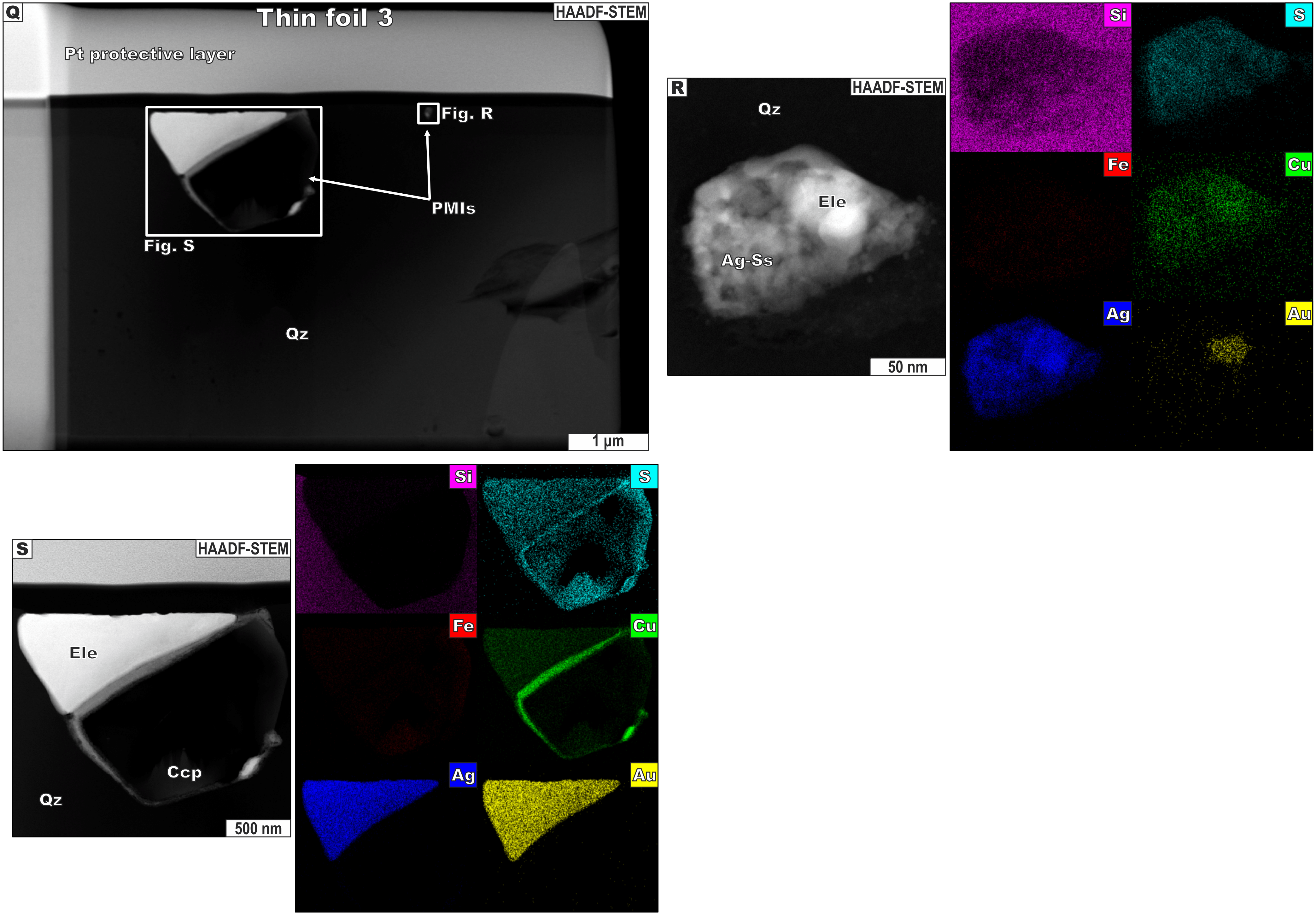

Supplementary Fig. 5. Q. High-angle annular dark-field (HAADF) image of thin foil 3. R-S. Scanning transmission electron microscopy (STEM) HAADF images and STEM compositional maps of polymineral inclusions (PMI) from thin foil 3. Key: Aca = acanthite, Ag-Ss = Ag sulfosalt, Ccp = chalcopyrite, Ele = electrum (Au-Ag alloy), Pea = pearceite, Plb = polybasite, Prs = proustite, Pyg = pyrargyrite, Qz = quartz.

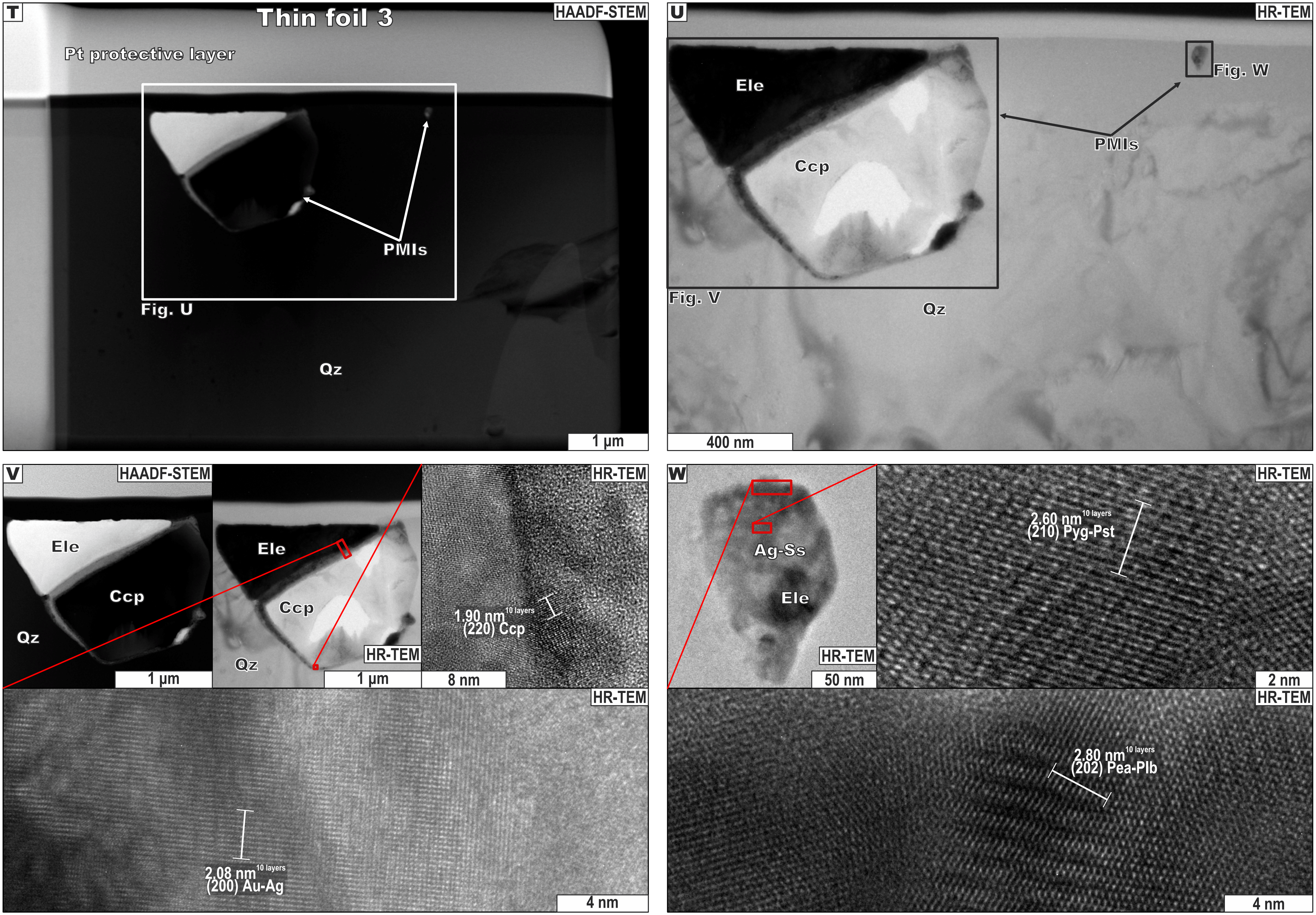

Supplementary Fig. 5. T. High-angle annular dark-field (HAADF) image of thin foil 3. U-W. Scanning transmission electron microscopy (STEM) HAADF images and high-resolution TEM images of polymineral inclusions (PMI) from thin foil 3. Key: Aca = acanthite, Ag-Ss = Ag sulfosalt, Ccp = chalcopyrite, Ele = electrum (Au-Ag alloy), Pea = pearceite, Plb = polybasite, Prs = proustite, Pyg = pyrargyrite, Qz = quartz.
