# Supplementary material for: Transient non-soluble noble metal transport in hydrothermal ore systems
Source: Nat Commun. 2025 Mar 13;16:2521. doi: 10.1038/s41467-025-57740-7 (PMC11906853; doi:10.1038/s41467-025-57740-7)
Supplement: Supplementary file 3 — Description of Additional Supplementary Files [file 41467_2025_57740_MOESM3_ESM.pdf]

<u>Description of Additional Supplementary Files</u>

Supplementary Data 1: Examples of hydrothermal ore deposits with mineralogical and textural features suggestive of precious-metal-rich melts.

Supplementary Data 2: Physicochemical features of polymineral inclusions (PMI).

Supplementary Data 3: Calculations of fluidiation velocity, settling velocity, pressure gradient, fluid viscosity, and fluid density.
